# The Black Death in Florence, Translated from the Italian of Boccacio

**Published:** 1847-09

**Authors:** Wm. M. Boling

**Affiliations:** Montgomery, Alabama


**Art. II.**—*The Black Death in Florence*, **translated from the Italian of Boccacio.**
**By Wm. M. Boling, M.D., of Montgomery, Ala.**

The years of the fruitful incarnation of the Son of God had reached the number of 1348, when in the famous city of Florence,—beautiful beyond any other of Italy,—happened the deadly pestilence, which,—through the operation of superior agencies, or for our iniquitous deeds, by the just wrath of God, in correction sent upon mortals,—some few years before, had commenced in oriental parts. Destroying innumerable lives, without interruption it spread from place to place, continuing its terrible march towards the occident. Neither judgment nor human foresight was of any avail; for the city was purged of much filth, by officers appointed for the purpose, and the entrance of all sick persons prohibited. Many good counsels were given for the preservation of health, and, to the same end also, humble supplications, not once, but many times, both in procession and otherwise, by devout persons, to God were made; as in the commencement of the spring of the year before named, horribly began its dolorous ravages; and in a miraculous manner too, to shew itself, and not as it had done in the east, with a discharge of blood from the nose—a manifest sign of inevitable death to whomsoever it occurred—but, in the commencement of this in men and women likewise, either in the groins or armpits, arose certain tumors—of which some were small, some larger, from the size of an egg to that of a common apple—which the vulgar called biles. From the two parts of the body already named, the aforesaid mortiferous biles indifferently to all parts, began to spread. And subsequently from this, the character of the aforesaid infirmity, changed into black or livid splotches, which in many appeared on the arms and thighs, and indeed on every other part of the body. In some they were large and sparse, in others minute and close together, as the biles at first had been; and also as these had been, were they, a certain indication of death to whomsoever they occurred.

For the cure of the disease, neither the counsel of the physician, nor the virtue of any medicine seemed of value or advantage. On the contrary, either that the nature of the disease would not admit of cure, or that the physicians, (of whom, besides the scientific, as well females as males, without having ever had any medical learning, the number became very great), ignorant of its true nature, were consequently incapable of treating it properly; not only did very few recover, but rather almost all, within the third day of the appearance of the symptoms just named, some sooner, others later, without any fever or other accident, died.

This pestilence was of greater power too, because, from the sick, to the well who had communication with them, it spread, not otherwise than as fire among dry or greasy substances, piled closely together. Further, also, the infection had this power, that, not only to talk to or approach the sick, caused the well to become sick, or was the occasion of their common death; but likewise to touch the clothes or any other thing which had been used or touched by the sick, seemed to communicate the disease to whoever touched them. A marvellous thing I am going to relate, and which if it had not been seen by the eyes of many and my own, scarcely would I believe it, much less dare to write it, however worthy of credit the narrator might be. I say, then, that so strong was the quality of communication of the disease from one to another, that not only man to man, extended it, but what is much more remarkable, though many times observed, the touch of the things of one who had been sick, or who had died of the disease, by any other animal of a different species from man, not only contaminated it with the disease, but caused its death within a very brief period. An instance of this kind, which with others, (as I have before remarked) I observed with my own eyes, I will relate. The rags of a poor man who had died of the disease, having been thrown into the public street, and two hogs coming across them, and according to their custom, first rooting them about with their noses, and then taking them in their teeth and shaking them around their cheeks, in a short time after staggering about, both fell to the earth dead upon the rags, as if they had taken poison.

From these things, and many others of a similar character, or more remarkable, arose divers fears and fancies in those who yet remained alive; and inclined all to the same cruel determination, which was, to shun and fly from the sick, and the things which had belonged to them; and in thus acting, each one thought he would secure his own safety.

There were some, who thinking that to live temperately, and guard against every superfluity, would have much efficacy in preventing the disease, with their families collected and shut up in houses where none were sick, lived separate from all others, and using in all temperance only the most wholesome food and the finest wines, avoiding every luxury, not speaking to any one, or admitting news of the dead or dying, from without; with music, and such other amusements as they were able to procure, passed their time. Others of a different opinion, affirmed, that to drink freely, to feast, to go about singing and rejoicing, to satisfy the desires in every thing possible, and that which happened, to laugh and make merry, was a certain medicine against the disease. Accordingly, as they said, they acted in so far as they were able; night and day going, now to this tavern, now to that, drinking without will, and without measure; and more than this, whatever in the houses of others they found to their pleasure or taste, they made use of. This they were free to do, seeing that others also (careless of the future) had, like themselves, abandoned their affairs, and hence the most of the houses had become common; and thus the stranger acted in any he entered as might have done the proper master himself; and pursuing this bestial course, they fled invariably, with all their power from the sick.

In this great affliction and misery of our city, the revered authority of law, Divine as well as human, was prostrate as it were and null; for the ministers and executors of these, as other men, were either all sick or dead, or so destitute of servants, that they were incapable of performing any of their duties, and consequently each person was permitted in all things, to pursue his own inclinations.

Many others pursued a middle course, between that of the two parties already named; not restraining themselves so much as the first in regard to diet, nor yet indulging so freely in wine and other excesses as the second, but of each used a sufficiency to satisfy the appetite. Nor did they shut themselves up in houses, but went around, carrying in their hands, some, flowers, some odoriferous herbs, and others various kinds of spices, of which they frequently inhaled the perfume, believing it to be an excellent thing with such odors to comfort the brain, seeing that the whole atmosphere seemed laden and stinking with the stench arising from drugs, and the dead bodies.

Some there were, of a more heartless disposition, (as perhaps it was the safest) who said that no medicine was better against the plague, nor so potent, as the flying from it; and impelled by this opinion, caring for nothing but themselves, numbers, both men and women, abandoned the city, their property, their friends, their own proper houses and dwelling places, and sought out others, mostly in the country; as if the wrath of God, to punish the iniquity of man, could not follow them with pestilence; but provoked against, intended to afflict only those who were found within the walls of the city, seeming to think, too, that none should remain, or that the last hour of all such had come.

All did not die of any of these different classes, neither did all of any one of them escape. On the contrary, many of each of them growing sick, and—having in every place themselves when they were well, set the example to those who yet remained well,—abandoned by all, died. Not only did one citizen avoid another, neighbors become regardless of each other, relatives visit each other never or seldom, and then without approaching closely; but, with such alarm had this tribulation filled the breasts of both men and women, that one brother abandoned another, the uncle the nephew, the sister the brother, and often the wife the husband; and what is still more astonishing, and scarcely to be credited, fathers and mothers refused to visit or serve their children, as if they were not theirs. On this account, to those who fell sick,—of whom the number both male and female was incredible—no other assistance remained, but from either the charity of friends—and of these there were few—or the avarice of hirelings, who served on their own terms, and for exorbitant wages; and notwithstanding this, few could be procured; and these, both men and women, were so ignorant, and the most of them so unaccustomed to such service, that they were of no other use than to hand to the sick such things as they asked for, or to watch when they died; and in such service, many of them, with the profits of their hire, lost their lives also.

From the sick being thus abandoned by neighbors, relatives, and friends, and from the scarcity of nurses, originated a custom, before unheard of, that, ladies, however beautiful, elegant, or noble of birth, being sick, were willing to be nursed by men, young or not, and to them without shame would expose their persons as they would have done to women; so much did the necessity of their condition overcome their native modesty, and this perhaps afterwards was the cause of less virtue in those who recovered.

Many died who perhaps if they had been assisted would have recovered; hence from the want of suitable attention—which the sick could not procure—and the violence of the pestilence itself, so great in the city was the multitude of those who by day and by night died, that it was wonderful to hear of, much less to behold; and as if from necessity things contrary to established custom among the citizens originated, with those who yet survived. It was the custom before, as we see practiced at the present day, for the female friends and neighbors, to collect in the house of the dead, and there to lament with and console the bereaved; and on the other part also, before the house with the male relatives united the neighbors and other citizens, and according to the rank of the dead came the clergy; and upon the shoulders of equals, with funeral pomp, chants, and lighted tapers, to that church which before death had been selected, the corpse was carried; which things, after the pestilence commenced to rage with violence, entirely, or in a great measure, ceased, and others, altogether new, in their place were adopted; because, not only did people die without this numerous collection of females around them, but many there were who passed from this life without a witness, and few there were to whom the pious lamentations and bitter tears of kindred were conceded. On the contrary, in place of these, were found laughter, jesting, and hilarity, which course the females, setting aside all womanly piety, more especially pursued, believing it conducive to health. There were few whose remains were accompanied to the church by more than one-tenth or twelfth of their neighbors; and not the honorable and valued citizens—but a set of grave-diggers, newly sprung up from the lower ranks, supporting the hearse—performed this delicate service; and with hurried steps, not to that church which before death had been selected, but to the nearest, in most instances, they carried it, behind four or five priests—who with the aid of the aforesaid grave-diggers —without fatiguing themselves with a long or solemn service, in the first unoccupied grave they found, hastily placed the corpse.

Among the lower ranks, and perhaps in a large portion of the middle, did the greatest misery prevail; because these, mostly, either by hope or poverty retained, remaining in their own houses and neighborhoods, by thousands a day sickened, and, being neither nursed nor aided with any thing, as it were without any attention, died. Many there were, who in the public streets, both by day and by night, breathed their last; and many also who died in their houses rather from the odor arising from their putrifying bodies ,than otherwise, gave evidence to their neighbors, that they were no more; and with dead bodies houses everywhere seemed filled.

There was among neighbors principally a course of this kind pursued:—Moved, not less by the fear that the corruption of the bodies might prove noxious, than by charity for the departed—by the assistance of porters, when these could be procured—they dragged the bodies of the dead from their houses, and placed them before the doors, where, in the morning especially, by the passer-by, they might be seen without number. They also caused hearses to be brought, but so numerous were the dead, that from a deficiency of these, the bodies were placed upon any kind of boards that could be procured. Nor was there but one only that carried two or three at once, nor did this happen only a single time, but many might be encountered, which bore the wife and the husband, two or three brothers, or the father and son, or similar burthens. And many times it happened, that two priests, going with the cross in front of one hearse, three or four other hearses, by the bearers, were added to the procession; and instead of one body which they supposed they had to inter, found seven or eight, and sometimes more. Nor were they with tears, or tapers, or company honored; on the contrary, things had arrived at such a pass, that men who died, were regarded, or cared as little for, as goats would be at the present day.

For the interment of the great number of bodies, which every day and almost every hour were carried to the churches, the consecrated ground not being sufficient, to give to each an appropriate place according to ancient custom, after every part was filled, deep pits were dug in the cemeteries in which in hundreds were placed those that followed, piled, as merchandise in vessels, layer upon layer, covered lightly with earth, till they reached the top.

And that to the end, that in regard to our passed miseries, in every particular I need not confine myself to the city—I will say, that the pestilence, passing through this, did not spare therefore, in any respect, the surrounding country, where (setting aside the castles, in which on a smaller scale, the same took place as in the city) in the scattered villages, the poor and miserable laborers, and their families without the care of the physician or the aid of nurses, in the roads, in the fields, and in their houses, by day and by night, without distinction died, not as human beings, but as animals. On which account, these in regard to ancient custom, like the citizens, became indifferent, caring for neither their property nor their business. On the contrary, all, as if the day had arrived on which they might expect death, neglected their flocks, the cultivation of the soil, and the gathering of its products; seeming anxious only to make way with what they had already laid up. Wherefore it happened that the cattle, the asses, the sheep, the goats, the swine, the poultry, and even man's faithful servant, the dog—neglected at home—wandered at liberty through the fields, where the grain, abandoned, lay cut, but ungathered. And many like rational beings, after having fed well through the day, at night returned, well filled, to their proper sheds, without the care of the herdsman.

But leaving the country, and returning to the city, what further can be said of the extent of the distress, than that such and so great was the cruelty of Heaven, and perhaps in part of man too, that between March and July, from the deadly nature of the malady, and from many of the sick being badly nursed, or—through the dread which possessed the well—abandoned altogether in their distress, over a hundred thousand human beings, it is believed for certain, died within the walls of the city of Florence; though perhaps before the deadly visitant, it may not have been supposed to contain so many. Oh, how many magnificent palaces, how many beautiful houses, how many noble habitations remained empty, even to the last servant! Oh, how many ancient families, how many ample inheritances, what amassed wealth, remained without a lawful inheritant! How many brave men, how many beautiful ladies, how many gay youths, who, not by ordinary persons, but by Galen, Hippocrates, or Æsculapias would have been pronounced well, at noon dined with their relatives, companions, and friends, and in the evening after, in the other world, supped with the departed!

Montgomery, Ala., April 8th, 1847.

